# Endovascular treatment of congenital descending aorta coarctation complicated by multiple tandem spinal artery aneurysms: a case report and literature review

**DOI:** 10.3389/fsurg.2026.1771342

**Published:** 2026-02-23

**Authors:** Chao Dang, He Hou, Jian-Chun Sheng, Kun-Yuan Zhu, Li-Gang Chen, Ting-Zhun Zhu, Guo-Biao Liang

**Affiliations:** 1 Department of Neurosurgery, General Hospital of Northern Theater Command, Shenyang, China; 2 Graduate School of Dalian Medical University, Dalian, China

**Keywords:** congenital aortic coarctation, endovascular treatment, multiple tandem aneurysms, spinal artery aneurysm, subarachnoid hemorrhage

## Abstract

**Background:**

Spinal artery aneurysms are a rare type of aneurysm, and their diagnosis and treatment are challenging. In this case report, we describe a patient in whom congenital descending aorta coarctation was complicated by subarachnoid hemorrhage secondary to the rupture of a multilevel spinal artery aneurysm, which was treated with endovascular surgery.

**Case description:**

A 54-year-old man presented with head and neck pain, nausea, and vomiting. He had congenital descending aorta coarctation, which was untreated. Imaging was notable for subarachnoid hemorrhage (SAH) (Modified Fisher grade 3), severe congenital descending aorta coarctation, and multiple aneurysms of the anterior spinal artery and left middle cerebral artery (MCA). One month after external ventricular drainage (EVD) and lumbar drainage (LD), his clinical status gradually stabilized. Owing to the poor general condition and the presence of multiple aneurysms, open surgery was deemed unsuitable, and endovascular treatment was performed. The patient had achieved partial recovery at the15 days operative follow-up.

**Conclusion:**

This case indicates that foramen magnum SAH with no identifiable source on conventional DSA warrants further investigation via cervical and thoracic myelography, CTA or MRI. Endovascular treatment may be considered for patients with multilevel spinal artery aneurysms and poor surgical candidacy due to frailty.

## 1 Background

Coarctation of the aorta (CoA), a congenital disease, accounts for 6%–8% of congenital cardiovascular diseases (1). It is characterized by localized stenosis of the aortic lumen, with compensatory dilatation of the vascular lumen proximal to the stenosis due to elevated intraluminal pressure. This stenosis causes a significant change in the original hemodynamics, leading to the development of new collateral circulation to maintain distal tissue perfusion (2). Spinal artery aneurysm (SAA) is a rare disease associated with vascular abnormalities, including arteriovenous malformations and CoA (3). Such abnormalities can lead to significant alterations in local hemodynamics, potentially affecting tissue perfusion. Owing to the scarcity of reported SAA cases, its epidemiology and natural history remain poorly defined (4). The occurrence of multiple SAAs secondary to CoA is exceptionally rare (5). It is estimated that less than 1% of patients with SAH have intraspinal lesions, and the clinical manifestations of SAH often include back and neck pain (6). The combination of these conditions poses a diagnostic and therapeutic challenge. We report a case in which congenital descending aorta coarctation complicated by SAH secondary to rupture of a multilevel SAA was successfully managed via endovascular treatment.

## 2 Case report

**Figure 1:**
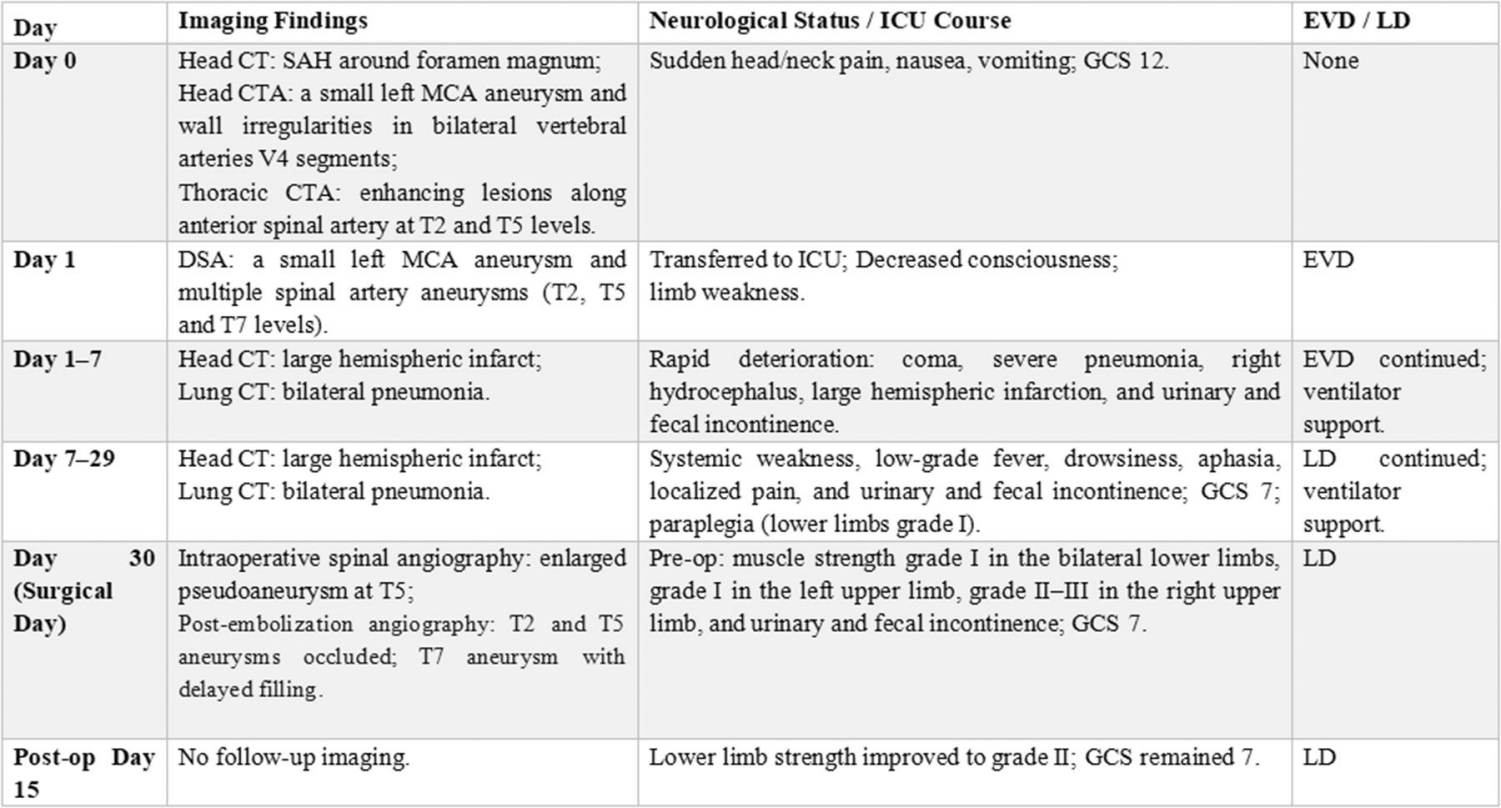
Timeline of the case report.

A 54-year-old man was first admitted to the hospital with sudden head and neck pain accompanied by nausea and vomiting for 3 h (Figure 1). He had congenital descending aorta coarctation, which was left untreated. Laboratory investigations revealed negative results for antinuclear antibodies, complement components, rheumatoid factor, and immunoglobulins and the patient underwent surgical treatment for a left middle cerebral artery (MCA) aneurysm (with details unspecified). He also had a long history of hypertension and smoking. An emergency physical examination after admission revealed that the patient was in a state of clouding of consciousness and lethargy, opened his eyes to verbal stimuli, and had decreased muscle strength in all extremities. The patient had nuchal rigidity, the Kernig sign (+), and the Brudzinski sign (+), and his Glasgow Coma Scale (GCS) score was 12 points (E3V5M4).

**Figure 2:**
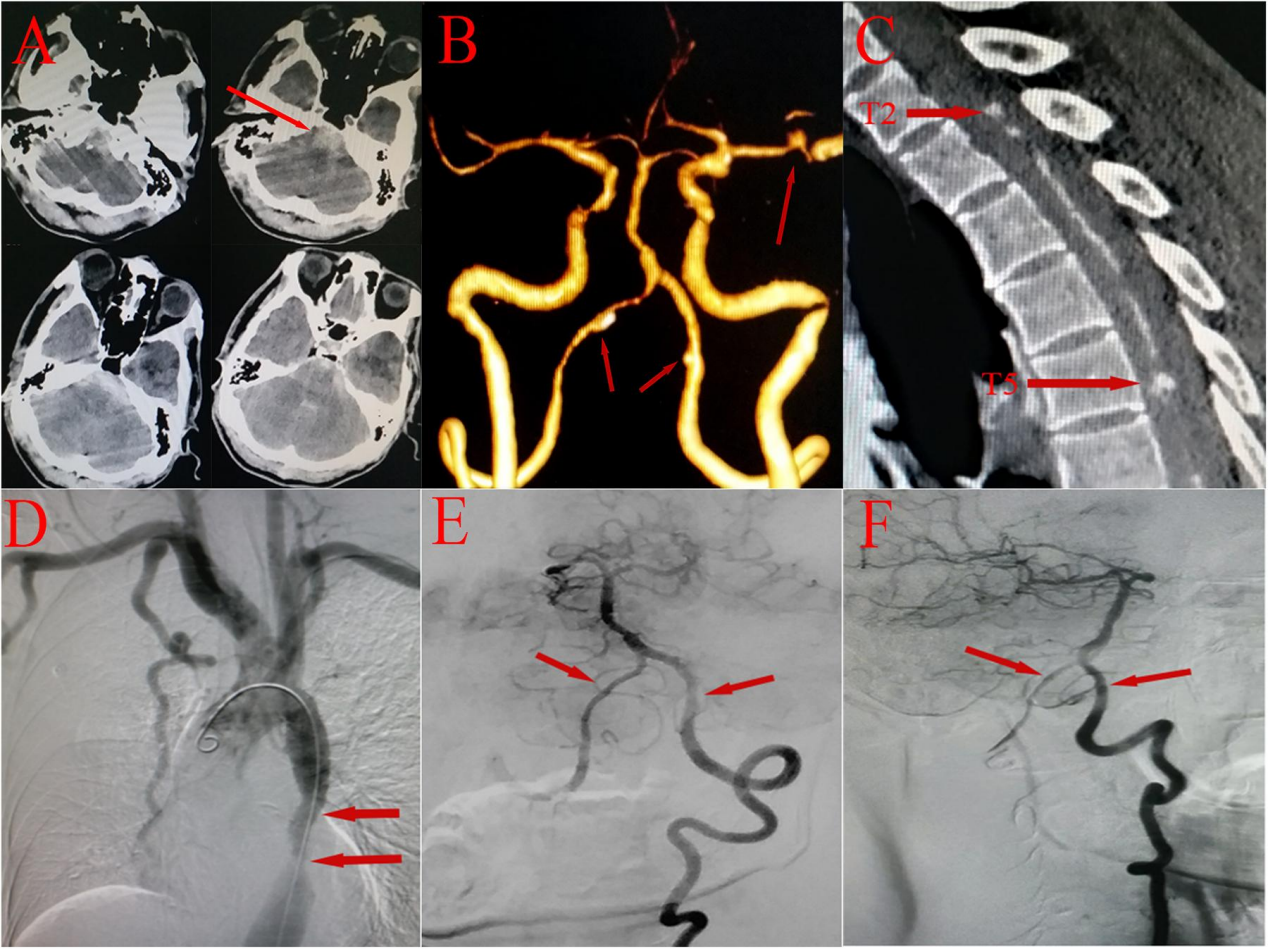
**(A)** CT scan of the skull base showing a high-density shadow around the foramen magnum and ambient cistern (red arrow). **(B)** Head CTA image demonstrating a left MCA aneurysm approximately 3 mm*2 mm in size and wall irregularities in the V4 segments of both vertebral arteries (red arrows). **(C)** Thoracic CTA image revealing multilevel circular enhancement lesions in the area of the anterior spinal artery (red arrows). **(D)** Aortic arch angiography revealing severe congenital descending aorta coarctation (red arrows). **(E,F)** Anteroposterior and lateral DSA showing no significant abnormalities in the V4 segments of either vertebral artery (red arrows).

**Figure 3:**
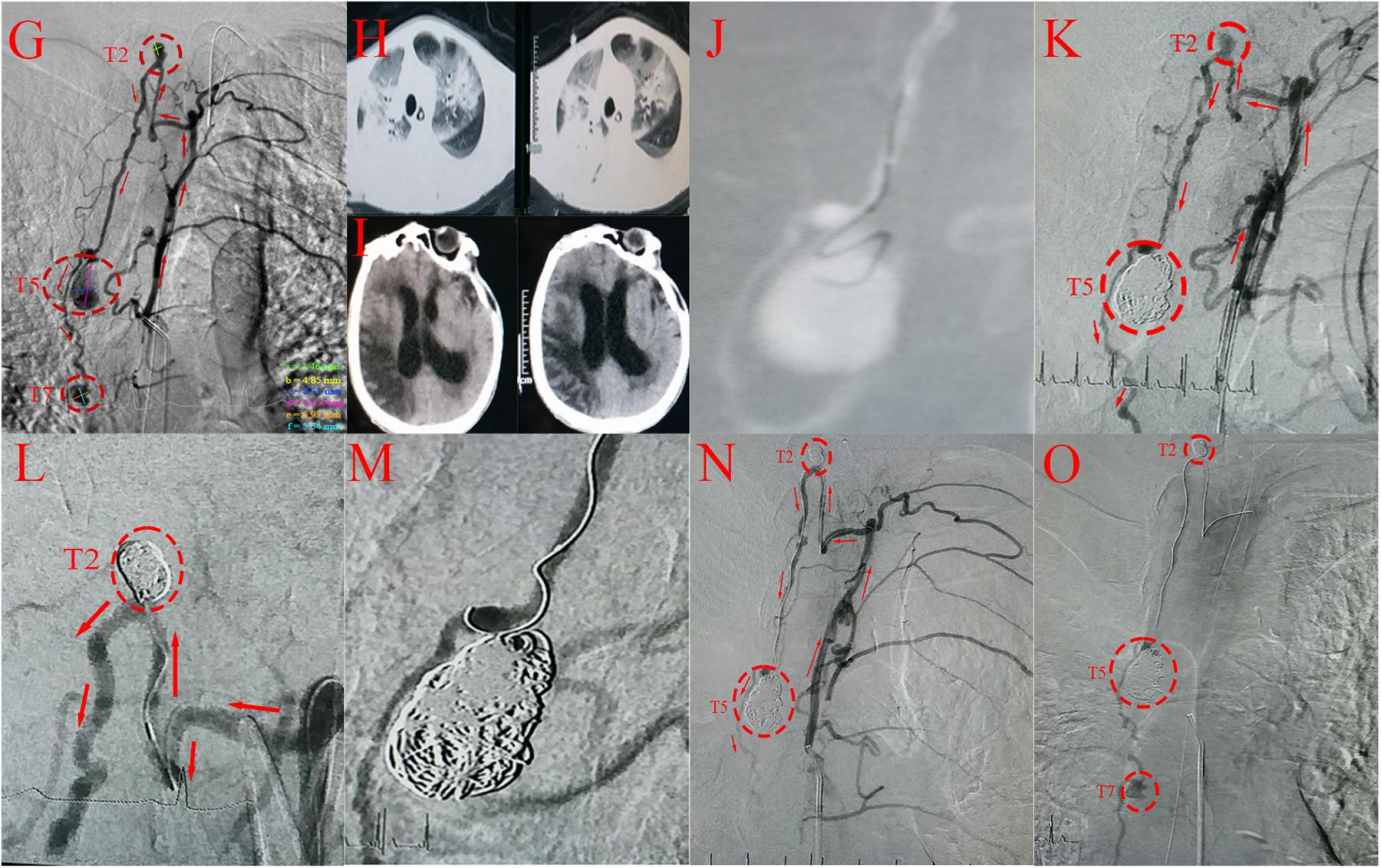
**(G)** spinal angiography confirming multiple tandem aneurysm-like structures in the spinal artery at the T2, T5 and T7 vertebral levels, with sizes of 3.5*4.9(saccular), 8.3*14.6(pseudoaneurysm) and 6.9*5.5(saccular) mm (red circles), and feeding artery (red arrows). **(H)** Lung CT revealing bilateral pneumonia. **(I)** Head CT showing a large area of infarction in the right cerebral hemisphere. **(J)** Spinal cord angiography confirming a pseudoaneurysm at the T5 level. **(K,L)** Embolization of T2- and T5-level aneurysms with coils (red circles). Red arrows represent the feeding artery. **(M)** Multiple microguidewire attempts during T7-level aneurysm embolization failing to achieve stable positioning. **(N,O)** Spinal cord angiography demonstrating that the aneurysms at the T2- and T5-level aneurysms were completely embolized (red circles) Red arrows represent the feeding artery. Delayed contrast opacification of the T7-level aneurysm during the late arterial phase suggesting a low rupture propensity.

Auxiliary investigations commenced with an emergency head computed tomography (CT) scan, which revealed high-density shadows around the foramen magnum and ambient cistern, consistent with SAH (Figure 2A). The patient subsequently underwent CT angiography, which revealed a small aneurysm in the left MCA and wall irregularities in the V4 segments of bilateral vertebral arteries (Figure 2B). However, based on the hemorrhage localization observed on the CT scan and the symptoms presented by the patient, we propose that the MCA aneurysm may not have been the lesion responsible for disease onset and suspect that the SAH may have been caused by intraspinal vascular disease. In support of this speculation, thoracic CTA revealed multilevel intraspinal contrast-enhancing abnormalities along the course of the anterior spinal artery at the T2 and T5 levels (Figure 2C). Finally, digital subtraction angiography (DSA) indicated severe stenotic coarctation of the descending aorta (Figure 2D), and the V4 segments of both vertebral arteries were normal (Figures 2E,F). Superselected spinal artery angiography confirmed multiple tandem SAAs at the T2, T5, and T7 levels (Figure 3G), which is consistent with the symptoms and other imaging findings. Considering the history of CoA, we speculate that CoA may have altered the hemodynamics of the spinal arteries, leading to increased intravascular pressure, which could have resulted in aneurysm formation and subsequent rupture, causing SAH. The patient was diagnosed with SAH (Modified Fisher grade 3), severe congenital descending aorta coarctation, and multiple aneurysms of the anterior spinal artery and left MCA.

Shortly after admission, the condition deteriorated rapidly manifesting sequentially as a coma, severe pneumonia, right-sided hydrocephalus and a large cerebral infarction in the right cerebral hemisphere (Figures 3H,I). To prevent re-rupture of the spinal aneurysm and stabilize the patient's vital signs in preparation for surgery. He received ventilator-assisted breathing and EVD/LD for symptomatic management. The EVD and LD were employed to relieve elevated ICP and the resulting hydrocephalus from SAH. After one month of therapeutic intervention, the vital signs stabilized, but his overall condition remained critical, characterized by systemic debilitation, low fever, phlegm, clouding of consciousness, aphasia, and pain localization. Neurological examination revealed severe paresis (muscle strength grade I in the bilateral lower limbs, grade I in the left upper limb, grade II–III in the right upper limb, and urinary and fecal incontinence), and his GCS score was 7 points. Given the poor physical condition and the presence of multilevel SAAs, open surgical intervention was not optimal because of the prohibitive perioperative risks. After the surgical options and risk benefit profiles were discussed with the family, endovascular treatment under general anesthesia was pursued.

**Figure 4:**
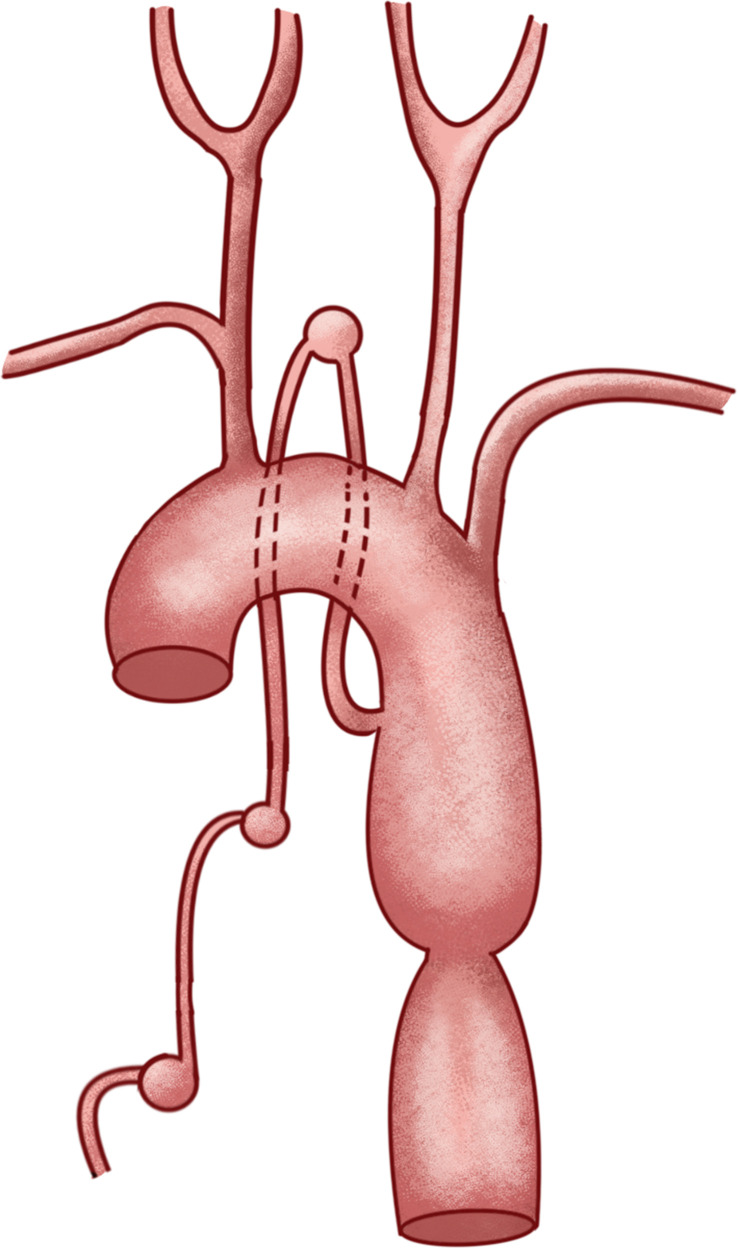
Anatomical diagram.

Surgical puncture of the right femoral artery of the patient was performed. A standard intravenous bolus of heparin (2,000 U) was administered after femoral access to achieve systemic anticoagulation. Intraoperative angiography revealed that the volume of the aneurysm at the T5 level was significantly greater than that before, and the aneurysm was considered a pseudoaneurysm (a responsible lesion) (Figure 3J). All the SAAs were supplied by the same radiculomedullary artery. The schematic depicts the specific anatomical structures (Figure 4). Endovascular treatment was performed in a staged manner: A 5F single-curved angiographic catheter (SCW Medicath Ltd, China）was placed through the Y valve and carefully advanced under fluoroscopy to cross the CoA under the roadmap. An Echelon 10 microcatheter (Micro Therapeutics Inc. dba ev3 Neurovascular Company, USA) was navigated over a Synchro 14 microguidewire (Stryker Neurovascular, Fremont, CA, USA) to selectively catheterize the T5-level pseudoaneurysm. Initial coil embolization of the T5-level pseudoaneurysm was successful, followed by successful embolization of the T2-level aneurysm (Target Coils, Stryker Neurovascular, Fremont, CA, USA) (Figures 3K,L). The T7-level aneurysm was ultimately not treated because excessive vessel tortuosity prevented microcatheter placement and caused significantly reduced blood flow at that location (Figure 3M).

Postoperative angiography immediately revealed complete occlusion of the T2- and T5-level aneurysms with no contrast filling and preserved patency of the spinal artery and parent artery. Delayed contrast opacification of the T7-level aneurysm during the late arterial phase suggested a low rupture propensity (Figures 3N,O), prompting the decision for secondary treatment. Postoperative intensive care, including back tapping and suction, resulted in controlled pneumonia and subsequent systemic improvement. The muscle strength in the bilateral lower limbs recovered to Grade II within 15 days after surgery.

## 3 Discussion

CoA is characterized by a narrow funnel-shaped constriction that may occur in any part of the aorta. Epidemiological studies have reported a prevalence of 0.047%–0.384% among live-born neonates (7, 8). Vincent reported the first angiographically revealed rupture of an anterior SAA in 1981. SAA, a rare category of spinal vascular pathology, accounts for only 1 case per 3,000 cases of spinal cord angiography (5, 9). It is generally associated with CoA, spinal arteriovenous malformations, connective tissue disease, vasculitis and other diseases (10, 11). SAAs demonstrate a female predominance (1.11:1), with a mean age at diagnosis of 38.1 ± 21.5 years, whereas multifocal presentations are exceptionally rare (5). The epidemiological profiles, optimal therapeutic strategies, and prognoses of patients with SAAs remain poorly understood (11).

The pathogenesis of CoA remains unclear. CoA not only alters hemodynamics in the descending aorta, causing significant dilation of the anterior spinal artery and the development of abnormal collateral circulation but may also induce symptoms of spinal cord compression within the confined spinal canal. Concurrently, the associated hemodynamic stress weakens the vascular wall, predisposing patients to aneurysm formation (12). In a retrospective analysis of 57 reported SAA cases, three patients (5.3%) demonstrated aneurysmal formation, which was definitively attributed to CoA (3). SAAs may also develop secondary to vascular wall thinning caused by connective tissue disorders, vasculitis, or infection. Notably, most SAAs resulting from inflammatory injury typically regress following immunosuppressive therapy (10, 13). The spontaneous regression of hemodynamically-induced SAAs is characterized by thrombosis within the dissecting aneurysm during endothelial healing—a process that may be facilitated by the slow flow in their small-caliber parent arteries (13, 14). SAAs differ from intracranial aneurysms in terms of hemodynamics, as spinal cord blood vessels are low-flow arteries; thus, the majority of SAAs do not occur at vessel branches. Hemodynamic alterations caused by CoA and its fragile collateral circulation increase the probability of SAH (5). In summary, CoA is not only an etiologic factor in SAA formation but also a critical trigger for its rupture and subsequent SAH. This understanding may be helpful for the diagnosis of SAH and has important diagnostic implications for patients with similar neurovascular conditions.

Many SAAs may be asymptomatic and undiagnosed, with the majority of SAAs diagnosed only after rupture with hemorrhage, contingent upon the manifestation of clinical symptoms (15). Consequently, the true incidence of SAA is likely substantially underestimated in clinical practice. The clinical manifestation of hemorrhagic spinal vascular lesions is characterized by a distinct temporal progression: sudden-onset back or neck pain accompanied by neurological deficits (including symptoms of sensory and motor changes in the bilateral lower limbs), which may subsequently present as intractable headache and classic signs of meningeal irritation (4). The presence of one or more of these symptoms in a patient with SAH should raise clinical suspicion for spinal vascular pathology, with the most frequent involvement of cervical and thoracic regions (16). SAA occurs most commonly in the anterior spinal artery but is not a frequent cause of spinal SAH (15). In our case, the initial imaging manifestation was SAH, which was detectable via CTA of the cervical and thoracic spinal regions. Aneurysms can also be detected via magnetic resonance imaging (MRI), manifesting as extramedullary, subarachnoid round masses deviating from the midline (17). With respect to the CoA diagnosis, in our case, a series of laboratory tests conducted for the detection of antinuclear antibodies, complement components, rheumatoid factor, and immunoglobulins were negative, ruling out immune-mediated arteritis. The presence of descending aortic stenosis shown by DSA, provided an additional basis for our diagnosis. Banna described a patient with simultaneous intracranial and spinal SAH caused by a ruptured SAA combined with CoA, as well as an unruptured intracranial artery aneurysm (18). The authors proposed that spinal angiography must be conducted for patients who develop fourth ventricular or posterior fossa hemorrhage. Spinal angiography is the gold standard for diagnosis and evaluation (11, 19). In our study, the MCA aneurysm was deemed an unlikely source of the acute SAH based on a clear mismatch between the hemorrhage pattern and the aneurysm location. The SAH was localized primarily to the foramen magnum and the ambient cistern on the initial CT. This is a posterior fossa distribution, which is highly atypical for a rupture of an MCA aneurysm, which typically results in hemorrhage concentrated in the Sylvian fissure. Furthermore, the clinical manifestation of hemorrhagic spinal vascular lesions is characterized by a distinct temporal progression: sudden-onset back or neck pain accompanied by neurological deficits. Finally, based on the findings from the thoracic CTA, spinal angiography, the history of untreated CoA and clinical symptoms, multiple SAAs were identified as the culprit lesion.

The optimal treatment strategy for SAAs is also controversial. At present, the following three primary approaches are employed: (1) surgical resection, (2) endovascular treatment, or (3) conservative treatment (20, 21). Surgical treatment involves clipping the aneurysm neck. Before this approach is selected, the location and size of the aneurysm, as well as whether the territorial artery can be preserved should be assessed (22). When endovascular treatment for SAAs is employed, it is important to consider that coil embolization at higher spinal levels may exacerbate compressive symptoms. With respect to conservative treatment, some consider compression of the spinal cord by an aneurysm or hematoma to be the only indication for intervention. While the possibility of spontaneous thrombosis in pseudoaneurysms can justify conservative treatment in frail patients, a ruptured spinal aneurysm mandates immediate surgical intervention (14). However, other factors, such as the size of the artery, the presence of distal blood flow, and the morphology of the aneurysm, also significantly influence treatment selection (23, 24). The prognosis for SAAs is generally poor, particularly in patients who present with neurological symptoms such as myelopathy, bladder dysfunction, weakness or sensory deficits. Additionally, patients with ruptured SAAs are more likely to experience neurological deficits, which further worsen their prognosis (5).

In our case, open surgery was deemed unsuitable because of the multiple segmental spinal aneurysms and the poor general condition. Compared with open surgery, endovascular treatment can prevent aneurysm rerupture while preserving the normal blood supply of the spinal cord. More importantly, it allows better management of severe pneumonia and other critical systemic conditions, and partial recovery of neurological function is also possible. In some patients with CoA, femoral artery cannulation may be challenging, and radial artery cannulation can be an alternative approach, as it prevents forcing the catheter through the stenotic arterial segment (25). In the present case, the patient exhibited paraplegia (Grade I muscle strength) preoperatively. Postoperatively, bilateral lower limb muscle strength improved to Grade II within 15 days, but the patient's GCS score was still 7 points (E2V1M4). There are some limitations in this study. Our intended post-procedural surveillance protocol involved spinal artery angiography at 1, 3, and 6 months to monitor the T7 aneurysm for stability. However, the patient's family declined this planned serial imaging follow-up due to the patient's overall debilitated condition and associated financial burdens. So this study did not obtain a clear long-term imaging follow-up.

## 4 Conclusion

(1) CoA with multiple SAAs is a rare clinical condition that typically presents as SAH resulting from SAA rupture, sudden back or neck pain, and neurological deficits. This disease is highly likely to cause disability in patients. Owing to its rarity, there are currently no established treatment guidelines for this condition. (2) For SAH in the foramen magnum region, if whole-brain DSA is negative, cervical and thoracic spinal angiography, MRI, or CTA should be performed to assist in the diagnosis. (3) Surgical treatment is prioritized for SAA, but endovascular treatment should be considered in the following situations: small aneurysms with minimal mass effects, AVM or AVF, poor overall condition and inability to tolerate surgical intervention, and multiple segmental aneurysms in the spinal cord. (4) These conclusions need to be verified in studies with larger sample sizes and longer follow-up times.

## Data Availability

The original contributions presented in the study are included in the article/Supplementary Material, further inquiries can be directed to the corresponding authors.
